# Hyperinsulinemic Hypoglycemia – The Molecular Mechanisms

**DOI:** 10.3389/fendo.2016.00029

**Published:** 2016-03-31

**Authors:** Azizun Nessa, Sofia A. Rahman, Khalid Hussain

**Affiliations:** ^1^Genetics and Genomic Medicine Programme, Department of Paediatric Endocrinology, UCL Institute of Child Health, Great Ormond Street Hospital for Children NHS, London, UK

**Keywords:** hyperinsulinemic hypoglycemia, congenital hyperinsulinism, glucose, insulin, K_ATP_ channels

## Abstract

Under normal physiological conditions, pancreatic β-cells secrete insulin to maintain fasting blood glucose levels in the range 3.5–5.5 mmol/L. In hyperinsulinemic hypoglycemia (HH), this precise regulation of insulin secretion is perturbed so that insulin continues to be secreted in the presence of hypoglycemia. HH may be due to genetic causes (congenital) or secondary to certain risk factors. The molecular mechanisms leading to HH involve defects in the key genes regulating insulin secretion from the β-cells. At this moment, in time genetic abnormalities in nine genes (*ABCC8, KCNJ11, GCK, SCHAD, GLUD1, SLC16A1, HNF1A, HNF4A*, and *UCP2*) have been described that lead to the congenital forms of HH. Perinatal stress, intrauterine growth retardation, maternal diabetes mellitus, and a large number of developmental syndromes are also associated with HH in the neonatal period. In older children and adult’s insulinoma, non-insulinoma pancreatogenous hypoglycemia syndrome and post bariatric surgery are recognized causes of HH. This review article will focus mainly on describing the molecular mechanisms that lead to unregulated insulin secretion.

## Introduction

Hyperinsulinemic hypoglycemia (HH) is biochemically characterized by the unregulated secretion of insulin from the pancreatic β-cells in the presence of low blood glucose levels. Under normal physiological conditions, β-cells synthesize, store, and secrete insulin in a precisely controlled manner so that the fasting blood glucose level is kept within a narrow range of 3.5–5.5 mmol/L (1).

The unregulated insulin secretion promotes glucose uptake into the skeletal muscles, liver, and adipose tissue, exacerbating the hypoglycemia further. Simultaneously, glucose production (via gluconeogenesis and glycogenolysis), lipolysis, and ketogenesis are all inhibited. In addition to the actions of insulin in inhibiting glucose production, there are also defects in the glucose counter regulatory hormones such as cortisol and glucagon (2–4). The lack of glucose (the brains primary source of fuel) accompanied by the deprivation of alternative substrates in the form of ketone bodies and lactate increase the risk of brain damage in these patients (5).

The genetic mechanisms that lead to HH involve defects in the key pathways regulating insulin secretion from pancreatic β-cells. These genetic defects perturb the normal physiological mechanisms of glucose-regulated insulin secretion, leading to inappropriate and unregulated insulin secretion. The unregulated insulin secretion leads to severe hypoglycaemia. The gene defects, which lead to congenital HH can be broadly categorised into two groups, channelopathies and metabolopathies.

The most severe forms of HH occur in the neonatal period (however, it can also present in the infancy, childhood, and in adults) and are usually congenital HH (CHH). HH may also occur due to secondary causes and these are summarized in Table 1 (6). The genetic causes of CHH are listed in Table 1.

**Table 1:** **An outline of different causes of HH**.

**Genetic causes of HH**
1. *ABCC8*
2. *KCNJ11*
3. *GCK*
4. *SCHAD*
5. *GLUD1*
6. *SLC16A1*
7. *HNF1A*
8. *HNF4A*
9. *UCP2*
**Secondary causes of HH (transient)**
1. Maternal diabetes mellitus (gestational and insulin dependent)
2. Intrauterine growth restriction
3. Perinatal asphyxia
4. Rhesus isoimmunization
**Metabolic causes of HH**
1. Congenital disorders of glycosylation
2. Tyrosinaemia type 1
**Syndromic causes of HH**
1. Beckwith–Wiedemann
2. Kabuki
3. Trisomy 13
4. Central hypoventilation syndrome
5. Leprechaunism (insulin resistance syndrome)
6. Mosaic Turner
7. Sotos
8. Usher
9. Timothy
10. Costello
**Miscellaneous causes of HH**
1. Postprandial HH
a. Insulin gene receptor mutation
b. Dumping syndrome
c. Non-insulinoma pancreatogenous hypoglycemia syndrome
d. Insulin autoimmune syndrome
e. Bariatric surgery
f. Insulinoma
2. Non-islet cell tumor hypoglycemia
3. Factitious hypoglycemia
4. Drug induced

**Figure 1:**
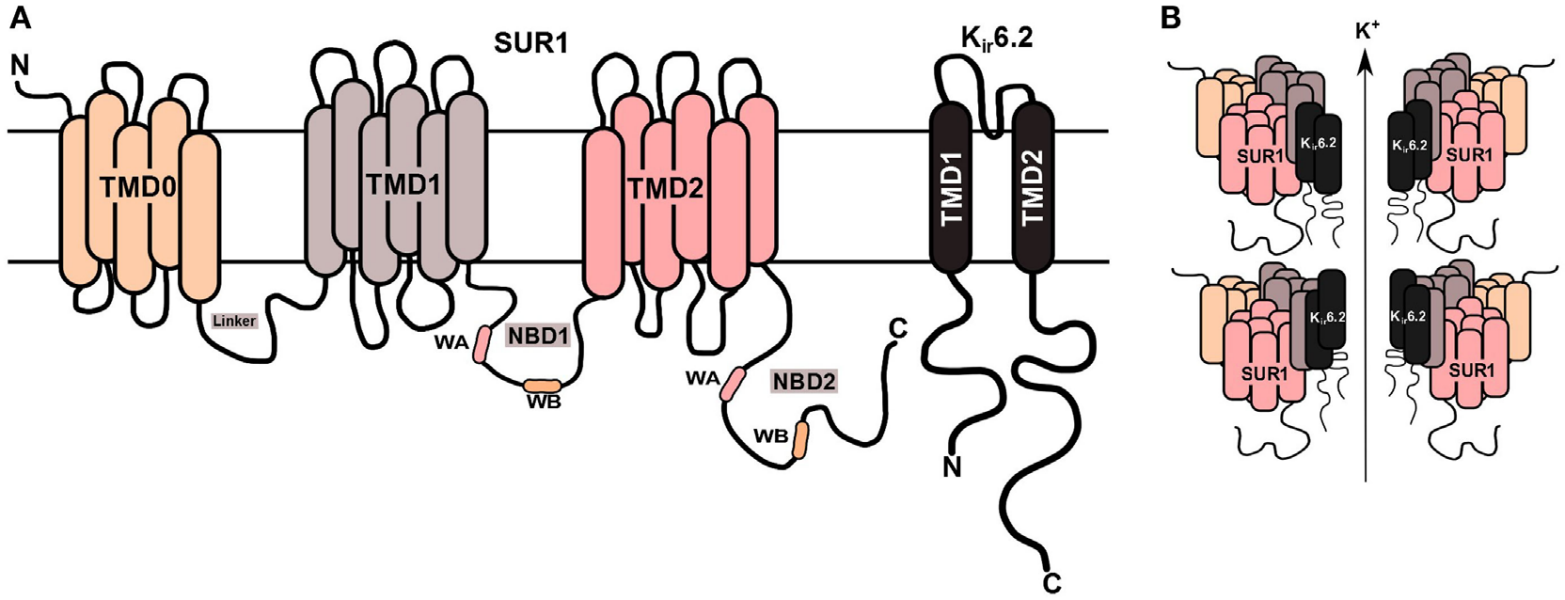
**K_ATP_ channel structure on β-cell membrane**. **(A)** The SUR1 subunit is made up of three transmembrane domains (TMD0, TMD1, and TMD2) and two nucleotide-binding domains (NBD1/NBD2), which face the cytoplasm. The NBD’s harbor the Walker A (WA) and Walker B (WB) motifs. K_ir_6.2 is the pore-forming subunit, containing two membrane-spanning domains, connected by an extracellular pore-forming region and cytoplasmic –NH_2_ and –COOH terminal domains. **(B)** Illustration of the predicted octameric structure of K_ATP_ channels, comprising four K_ir_6.2 and four SUR1 proteins.

The most severe forms of CHH are due to defects in the genes (*ABCC8* and *KCNJ11*), which encode for the sulfonylurea receptor protein (SUR1) and inwardly rectifying potassium channels (K_ir_6.2) proteins, respectively, of the potassium sensitive K_ATP_ channel (7, 8). These defects are referred to as channelopathies (9). The coassembly of four SUR1 and four K_ir_6.2 subunits forms a single ATP-sensitive potassium channels (K_ATP_) (Figure 1). The second category, metabolopathies, constitutes the majority of the other genes known to cause CHH.

In this review, we first review in detail the molecular basis of the ATP sensitive potassium K_ATP_ channel function, and then outline the molecular pathways that lead to CHH due to defects in these channels and second we describe the biochemical causes of HH due to defects in the non-K_ATP_ channel genes.

### Channelopathies: Background to ABC Proteins

The transportation of ions, molecules, and various other proteins across a membrane is of paramount importance to the functioning of all living cells. A simple example is of the *E. coli* cell which transports ~10^6^ glucose molecules per second into the cell in order to meet its physiological requirements (10). The movement of these essential (and non-essential) molecules is mediated by membrane-bound transporter proteins. There are 48 ATP-binding cassette (ABC) transporters present in humans (11). In the human genome, there are seven sub-groups of the ABC transporter family, involved in a diverse range of functions. This includes multidrug resistance, cholesterol and lipid transport, mitochondrial iron homeostasis, and also the ATP-dependent regulation of ion channels, which include the cystic fibrosis transmembrane conductance regulator and the sulfonylurea urea receptor. Many disease phenotypes arise as a result of mutations in the ABC genes, such as cystic fibrosis, diabetes, and CHH (12). The *ABCC8* gene stands for ATP-Binding Cassette, Sub-Family C, Member 8, and it codes for the SUR1 protein, which is a major example of an ABC protein.

All ABC proteins including SUR1 harbor two major domains, which consists of the transmembrane domains (TMD) and the nucleotide-binding domains (NBD). The TMD, situated in the cell membrane, is primarily composed of α-cell-helices. The NBD’s, however, are always found within the cytoplasm of the cell. A distinguishing feature of ABC proteins is the presence of highly conserved sequence motifs, such as the Walker A (WA) and Walker B (WB) motif. The former is a characteristic phosphate-binding loop, and the latter is Magnesium [Mg^2+^-binding site (13)]. Another striking feature is the Q-loop, which is positioned in between the WA and WB motif, known to associate with the gamma phosphate through a water bond. These three motifs combine together to form the NBD.

Other characteristic feature of ABC proteins is the “switch” region, which contains a histidine loop (H-Loop). It has been hypothesized that it polarizes the water molecule for hydrolysis. In ABC proteins, the variation arises within the TMD’s, which can vary among the different ABC proteins. This provides the membrane protein with its ability to sense changes in different nucleotides or other ions and compounds (12).

### The *ABCC8* Gene

The *ABCC8* gene is located within the human genome on chromosome 11p15.1. This gene has 39 exons (14), translating into either a 1581 or 1582 amino acid protein (15), which has a molecular weight of 176.9 kDa (16). The three transmembrane domains in SUR1 are TMD0, TMD1, and TMD2 (17). There are also two cytosolic nucleotide-binding domains (NBD1 and NBD2) within a single SUR1 transmembrane protein. SUR1 can combine ATP hydrolysis to transport substrates, however within the K_ATP_ channel complex it is primarily role is not to transport substrates; instead it regulates the activity of the K_ir_6.2 subunit. SUR1 also confers the K_ATP_ channel with its sensitivity and response to sulfonylureas such as glibenclamide (18) and channel activators such a diazoxide, which is commonly used to treat medically responsive CHH (19).

### SUR1: The Walker A, Walker B, and Nucleotide-Binding Domain

The WA and WB motifs contain the consensus sequence for nucleotide hydrolysis, making them one of the most prominent features of SUR1 proteins (20). A flexible loop structure is formed at the WA motif, which contains the sequence Gly–X–Gly–X–Gly. This is followed by a lysine residue, which is predicted to bind to the α-cell-phosphate of ATP. A hydrophobic β sheet is formed at the WB motif, which has an adjacent aspartic acid residue, and this has been postulated to interact with the Mg^2+^ ion of MgATP (13). It has been predicted that the WA and WB motifs in K_ATP_ channels are able to interact with the phosphoryl group, and then co-ordinate the Mg^2+^ in the MgATP complex (21). There is a third motif, which situated in between the WA and WB, and this region has been denoted as a “linker,” which transduces the effect of nucleotide binding and hydrolysis (22).

The WA and WB sequence motif is contained in both NBD1 and NBD2 of SUR1 (20), and this motif can sense the levels of intracellular nucleotides within pancreatic β-cells. The key nucleotide is ATP, which is produced in large quantities after a meal when cell metabolism is high. ATP has an inhibitory effect on K_ATP_ channels, although MgATP can have the opposite effect. The hydrolysis of MgATP is believed to involve a conformational change at the NBD’s, which in turn generates two nucleotide binding sites known an ATP-binding site 1 (ABS1) and 2 (ABS2), believed to mediate the hydrolysis of MgATP. Azido-ATP labelling experiments demonstrate that the binding of nucleotides to NBD2 stabilizes the binding of ATP to NBD1 (23). It has been shown that NBD1 and NBD2 can be coimmunoprecipitated (24). A soluble NBD1–GFP construct can be taken to the cell membrane by TMD2–NBD2 construct when they co-expressed in insect cells (25).

### The *KCNJ11* Gene

In the vertebrate genome, there are two genes coding for K_ir_ proteins, this includes the *KCNJ8* and *KCNJ11* genes which encode for the K_ir_6.1 and K_ir_6.2 proteins, respectively. The *KCNJ11* gene is also located on chromosome 11p15.1. However, in contrast to the *ABCC8* gene, the *KCNJ11* gene only contains 1 exon, which translates into a 390 amino acid protein with a molecular weight of 43.5 kDa (14). A distinctive feature of K_ir_ proteins is that they enable positive current to flow inwards into the cell, despite there being equal concentrations of K^+^ ions either side of the membrane. K_ir_6.2, however, has its own unique properties, the first being that it is not a strong inward rectifier (26); nevertheless its origins are also from a large super-family of K_ir_ proteins, namely K_ir_1–K_ir_7 (27, 28). In the K_ATP_ channel complex, the K_ir_6.2 proteins functions as the pore of the channel, allowing K^+^ ions to flow through. K_ir_6.2 is composed of two transmembrane domains, which are linked by an extracellular pore-forming region. The –NH_2_ and –COOH terminal domains are located within the cytoplasmic side of the protein.

## Histological Characterization of CHH

Three histological forms of CHH currently exist; focal CHH, diffuse CHH, and atypical CHH. Focal CHH is characterized by pathological pancreatic β-cells localizing to one or more specific locations in the pancreas. In contrast, in diffuse CHH, there are abnormal islet cells throughout the pancreatic tissue (29, 30). Although clinical presentation of both these types of CHH appears to be similar, their molecular mechanisms are different.

Atypical CHH are usually described as cases that are histologically difficult to categorize and are on general due to morphological mosaicism in a portion of the pancreas.

### Focal CHH

The focal lesion has abnormal islet cells, whereas the surrounding pancreatic areas seem histologically normal, but islets are reported to have small nuclei. The non-focal area islet cells have a reduction in cytoplasmic mass as well as diminished proinsulin production (30–33).

Focal CHH appears to be a result of two events; (1) a paternally inherited K_ATP_ channel mutation and (2) the loss of the corresponding maternal allele located within the focal area. This results in an imbalance in the expression of various imprinted genes. These include the expression of insulin-like growth factor 2 (*IGF-2*) (34) and tumor suppressor genes *H19* and cyclin-dependent kinase inhibitor 1C (*CDKN1C*). These genes have been shown to promote β-cell hyperplasia and are expressed paternally and maternally, respectively.

In a few cases, paternal allele duplication of chromosome 11 has also reported to cause focal CHH (35). However, the majority of focal CHH due to heterozygous paternally inherited mutation in the *ABCC8* or *KCNJ11* gene account for almost 30–40% of all CHH cases (36). The imbalanced 11p15 region interrupts pancreatic β-cell gene expression, which leads to focal islet hyperplasia.

Focal CHH is usually confirmed by a fluorine-18 dihydroxyphenylalanine-positron emission tomography (18F-DOPA-PET) scanning. This scan can identify the location of one or more focal lesions with diagnostic accuracy (37). Surgical resection of the lesion usually resolves HH.

### Diffuse CHH

The diffuse pancreas affects all the pancreatic β-cells, but islet morphology is preserved (32). Affected islets cells appear with large hyperchromatic nuclei and an abundant mass of cytoplasm (29–33). Patients with diffuse CHH either have a homozygous recessive or a compound heterozygous mutation in their K_ATP_ channel genes. This form of CHH accounts for 60–70% of all CHH cases. Patients are mainly medically unresponsive, and hence, require a “near total pancreatectomy.” However, this often leads to patients having life-long diabetes and exocrine insufficiency.

### Atypical CHH

Histologically, atypical CHH are categorized when the pancreatic morphology neither fits the focal nor diffuse CHH category (34, 38). The islets have been described to be enlarged or shrunken, and atypical CHH still remains poorly defined. Variability in management of atypical CHH occurs because some cases have been cured with a surgical resection while others also required medical therapy. 18F-DOPA-PET scanning usually used to recognize between focal and diffuse CHH has failed to identify atypical CHH (34).

To date, no associated mutations have been reported for atypical CHH, with the exception of one patient with an *ABCC8* nonsense mutation (p.Gln54X) (38). Other studies have described the heterogeneous expression of hexokinase 1 of the pancreatic β-cells in atypical pathology (39, 40). Furthermore, a recent study has also implicated an abnormal postprandial incretin response in two cases of atypical CHH of unknown genetic cause (40).

## K_ATP_ Channels and Insulin Secretion

In 1983, Noma and colleagues identified K^+^ currents from tissue extracted from heart muscle cells, when they were induced with metabolic poisons or under hypoxic conditions (41). During the period of 1983–1988, there was a vast amount of research done on understanding the basic electrophysiological properties of K_ATP_ channels. As a result, the expression of K_ATP_ channels has been observed in many tissues, including cardiac muscle, skeletal muscle, smooth muscle, pancreatic β-cells, and neurons (41–45). K_ATP_ channel currents have now also been identified in the rough endoplasmic reticulum of rat hepatocytes (46).

However, it was not until the mid-1990s that recombinant *in vitro* functional work had advanced in the field of K_ATP_ channels. It was soon realized that the lone expression of K_ir_ proteins was not enough to generate K_ATP_ channel currents in cells. This lead to the discovery of the SUR protein and the functional reconstitution of K_ATP_ channels (14). The coexpression of the proteins leads to the channel being sensitive to nucleotides and sulfonylureas.

Several studies carried out in isolated pancreatic β-cells have shown the functional significance of K_ATP_ channels as they play a major role in glucose homeostasis. These studies have used electrophysiological and radioactive techniques to elucidate the effect of sulfonylureas, potassium channel openers, and nucleotides on pancreatic β-cells (47–52).

**Figure 2:**
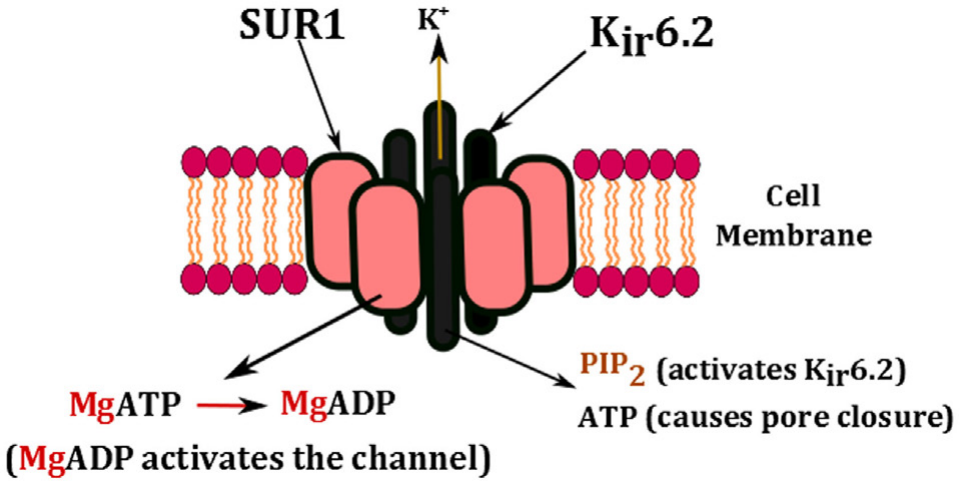
**Nucleotide regulation of K_ATP_ channels**. The channel is activated by the presence of PIP_2_ and the conversion of MgATP to MgADP. High concentrations of ATP block the pore and cause channel closure.

The key regulators of K_ATP_ channels are intracellular nucleotides such as ATP and ADP (Figure 2). This has a direct link to cellular metabolism as the production and consumption of ATP has a direct impact on the activity of K_ATP_ channels. A rise in the intracellular concentration of ATP changes the physical conformation of K_ATP_ channels from an open to a closed state. The closure of K_ATP_ channels in the β-cell membrane changes the resting membrane potential (−70 mV) from being hyperpolarized to being depolarized (−50 mV), which in turn triggers the activation of voltage-gated calcium channels (53). The influx of Ca^2+^ ions into the β-cell enhances the exocytosis of insulin, which ultimately aims to restore blood glucose levels (54).

### Gating Mechanisms in K_ATP_ Channels

The gating properties of K_ATP_ channels are affected by many different ligands. ATP has the ability to block the channel both in the presence and absence of Mg^2+^ ions, whereas PIP_2_ can activate the channel (Figure 2) (55–57). It has been suggested that there may be two gates in the permeation pathway. It has been observed that there is in fact ligand-independent gating, which indicates that the gate may be in the selectivity filter. Experimental observations suggest that K_ATP_ channels may harbor two independent types of gates in the permeation pathway (58). This includes:

(1) Ligand independent: fast gating, controlled by changes in membrane potential, this suggests that there is a selectivity filter, something which allows K^+^ through irrespective of all metabolic changes.
(2) Ligand dependent: slow gating, modulated by membrane PIP_2_ and nucleotides.

Experiments studying the kinetics of K_ATP_ channels reveal that un-liganded subunits are quite unstable. This means that subunits, which are not bound by either ATP or PIP_2_, are unstable, but once bound, they are mutually excluded. Hence, the subunits will be bound by one ligand or another at any one time.

Wild type (WT) K_ATP_ channel subunits are bound to PIP_2_ in the absence of ATP, which is why the channels are in the open state around ~90% of the time (59). But for the channel to be active, all four subunits must be in the open state. However, binding of ATP to the un-liganded subunit will trap it into the closed state. In WT subunits, the association constant for ATP is around ~10 μM. In energized cells, the concentration of ATP is two to three magnitudes higher; therefore, it is likely that each WT subunit is bound by ATP around ~90% of the time. Locking any one subunit in the closed conformation is enough to induce channel closure (60, 61). So apart from extreme conditions, the probability of the channel being in the open state is <0.1^4^, which means that without the stimulatory effects of Mg^2+^ nucleotides the channel will almost always remain closed. PIP_2_ has the effect of “pulling” the channels to open, whereas ATP does the opposite and tries to “push” the channels to close.

Previous studies have shown that ATP destabilizes the channel open state and overall it enhances channel closure (62–65). In earlier studies, it was understood that the interaction of one ATP molecule with the open state resulted in channel closure. However, there are four K_ir_6.2 subunits in every K_ATP_ channel complex, therefore there are four ATP inhibitory binding sites and so up to four ATP molecules can bind to the open state. The binding of four ATP molecules can either work together in concert to induce channel closure or they can act independently (66). To this date, the relationship between the number of ATP molecules bound and the closure of the K_ir_6.2 pore is not well understood. Hence, it is debatable on whether the K_ATP_ channel, which is an octameric complex, acts under a concerted gating mechanism using all four subunits, or whether there are transitional steps involving each subunit.

### Nucleotide Regulation of K_ATP_ Channels

The changing concentrations of ATP, ADP, GTP, and GDP regulate the opening and closing of the K_ATP_ channels. Adenine nucleotides, such as ATP and ADP, are able to interact with Mg^2+^ ions in a stimulatory manner; however, in the absence of Mg^2+^ ions, the non-hydrolytic binding of ATP takes precedence over K_ATP_ channels and promotes channel closure. Mg^2+^ ions are one of the most vital elements in the human body; hence, they are present at all times inside cells (67). Therefore, in order for the K_ATP_ channels to close, there must be significantly higher levels of ATP than MgADP.

MgADP can stimulate channel activity by direct binding, or by the binding and hydrolysis of MgATP (68). This can be seen in Figure 2. ATP binds to the cytosolic domain of K_ir_6.2. In a study by Craig et al. (66), they examined the mechanism of inhibition by ATP, and they achieved this by removing Mg^2+^ from the solutions, which enables them to study ATP interactions in isolation from nucleotides (66). The single channel kinetics of K_ATP_ channels show bursts of short openings and closings, which are separated by long, non-conducting interbursts intervals. Once ATP is added however, it causes a notable reduction in bursts duration, and a significantly longer duration of the closed states. Hence, ATP interacts with all these states, and ATP ends the burst state and aids the start of the long closed state.

Until 1997, it was widely accepted that the prime location for ATP inhibition was on the SUR1 subunit of the K_ATP_ channel complex, possibly within the NBD’s (69). However, the discovery that a truncated K_ir_6.2 protein could produce a homomeric channel (70), and function similar to a K_ATP_ channel, made it clear that the ATP inhibition actually affected the K_ir_6.2 subunit.

### K_ATP_ Channel Mutations Causing HH

**Figure 3:**
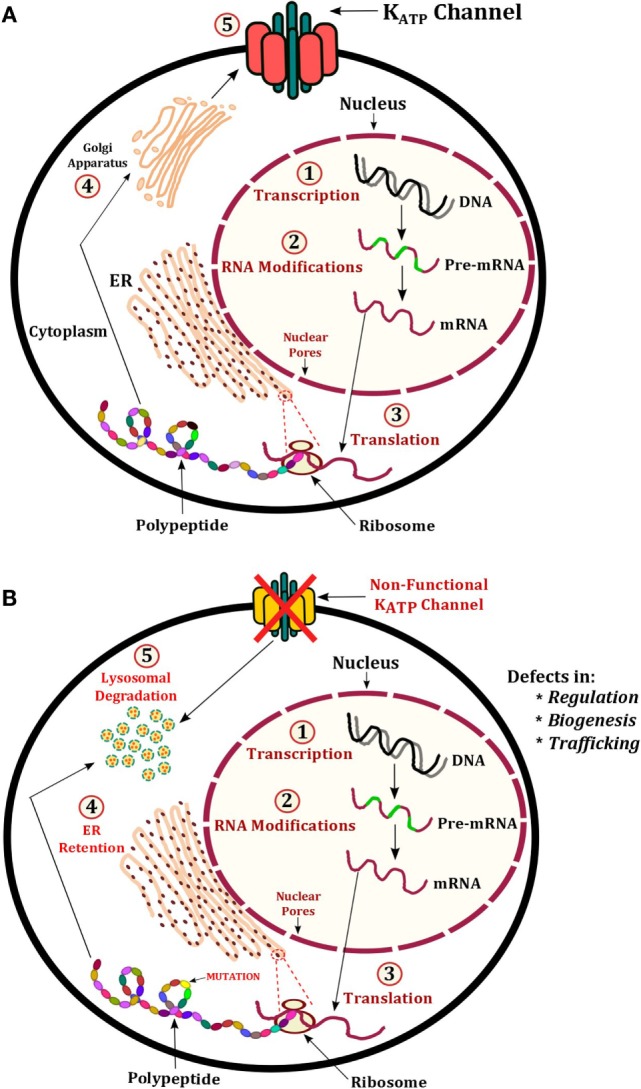
**Illustration of K_ATP_ channel protein production and regulation**. **(A)** Normal production of K_ATP_ channels involves transcription of *ABCC8* and *KCNJ11* to produce pre-mRNA, this undergoes modification to become mature mRNA. The mRNA exits the nucleus and is translated into a protein on ribosomes embedded on the ER. The polypeptide(s) fold into the tertiary structure and enter the Golgi apparatus for post-translational modifications. Vesicles containing the fully assembled K_ATP_ channel proteins are then expressed at the membrane. **(B)** Mechanisms of CHI include defects in regulation, biogenesis, and trafficking. In these cases, the defective K_ATP_ channel may undergo protein degradation in lysosomes.

Inactivating mutations in any region of the *ABCC8* and *KCNJ11* genes account for the vast majority of mutations known to date. Mutations in these genes cause the most severe form of CHH that is typically medically unresponsive to therapy with diazoxide. The condition presents in the neonatal period with severe hypoglycemia, which is difficult to control with oral feeds and will require the infusion of concentrated intravenous dextrose. Both recessive and dominant mutations can have a major negative impact on many aspects of K_ATP_ channel function, regulation, and processing. Ultimately, this leads to persistent depolarization of the pancreatic β-cell membrane, which leads to unregulated insulin secretion (71). A recessive mutation observed in >90% of Ashkenazi Jewish population is p.Phe1388del (*ABCC8*) (9), which is known to cause severe CHH (14, 15). The p.Val187Asp and p.Glu1506Lys mutations in *ABCC8* are largely found in the Finnish populations, which are also known to cause CHH. A schematic representation of known mechanisms of K_ATP_ channel defects has been shown in Figure 3. This section will describe some of these known mechanisms.

### Mechanism 1: Recessive K_ATP_ Channel Mutations

The membrane expression of K_ATP_ channels is vital to its role in maintaining the resting membrane potential of pancreatic β-cells and insulin secretion. In order for K_ATP_ channels to traffic to the plasma membrane, the SUR1 and K_ir_6.2 subunits must first assemble into their tertiary protein structure. This allows the endoplasmic reticulum retention signal known as RKR (Arg–Lys–Arg) present in both subunits to be shielded, enabling successful migration of the K_ATP_ channel complex from the endoplasmic reticulum to the plasma membrane (Figure 3) (72, 73). The following *ABCC8* mutations cause trafficking defects including: p.Arg1437Gln, p.Phe1388del, p.Arg1394His, and p.Leu1544Pro. The *KCNJ11* mutation p.His259Arg produces a non-functional K_ATP_ channel with impaired trafficking to the cell surface.

Within the SUR1 subunit, the RKR signal is located on the N-terminal of NBD2, and in K_ir_6.2, it is on the C-terminal. Removal of the last 36 amino acid residues in K_ir_6.2, which contains the RKR signal results in the membrane expression of a homotetrameric K_ir_6.2 structure, which has unique properties unlike that of K_ATP_ channels (70). There are prominent differences between WT K_ATP_ channels and homotetrameric K_ir_6.2 channels. For example, the half-maximal inhibition concentration (IC_50_) by ATP is ~10-fold higher in homotetrameric K_ir_6.2 channels compared to WT channels.

The SUR1 and K_ir_6.2 subunits of the K_ATP_ channel complex are sensitive to stimulation via Mg^2+^ bound nucleotides (MgATP, MgADP). A key function of the SUR1 subunit is to regulate the conductance of the K_ir_6.2 protein within the channel complex. Recessive mutations have been shown to affect the conductance of the K_ATP_ channel, and this includes the *ABCC8* mutations p.Thr1139Met and p.Arg1215Glu (74). These mutants display a loss of ADP-dependent gating, which causes constitutive inhibition of K_ATP_ channels by ATP. The p.Arg1420Cys is another *ABCC8* mutation, which generates a K_ATP_ channel that has a fivefold reduced affinity for ATP and ADP (75). In addition, the ability of MgATP and MgADP to stabilize ATP binding at NBD1 is also affected. The p.Arg1420Cys mutation acts as a hindrance to the transduction of a conformational change at NBD2 and thereby its ability to stabilize ATP binding at NBD1.

### Mechanism 2: Dominant Mutations in *ABCC8* and *KCNJ11*

The p.Glu1506Lys mutation was the first dominant mutation identified in the *ABCC8* gene, which caused CHH in seven related patients (76). The diagnosis was based on the presence of inappropriately high levels of insulin, non-ketotic hypoglycemia, and a raised demand for glucose in order to prevent hypoglycemia. Mutations in the *KCNJ11* gene causing diazoxide responsive CHH have also been reported. Patients with a dominant mutation in *ABCC8* or *KCNJ11* have a milder form of CHH and are mostly responsive to medical therapy (77), thus avoiding a pancreatectomy. The age of presentation is usually later than those with the recessive condition. Dominant mutations in *ABCC8* and *KCNJ11* demonstrate normal assembly with their respective WT partner and normal trafficking of assembled channels to the plasma membrane (78). However, dominant mutations causing diazoxide unresponsive CHH can generate K_ATP_ channels, which are retained in the endoplasmic reticulum.

### Functional Consequences of Dominant Diazoxide Responsive CHH

Thornton et al. (79) identified a novel tri-nucleotide deletion in the *ABCC8* gene, which caused the loss of serine 1387 (79). Experiments were conducted in COSm6 cells using human K_ir_6.2 and SUR1, which were transfected by electroporation. The rubidium (Rb^86+^) flux assay using metabolic inhibitors (2-deoxy glucose and oligomycin) and diazoxide revealed that when the p.Ser1387del mutation was expressed alone, it showed no response to metabolic inhibitors, or even to lower ATP concentrations, hence the mutation was disease causing. However, when the p.Ser1387del was expressed with WT SUR1, it generated the same level of activation as the pure WT channels. This is in contrast to the p.Glu1506Lys mutation, which only exhibited partial response to channel activators when expressed with WT SUR1 (76). The p.Ser1387del mutant, however, does reach the plasma membrane. Hence, the cause of CHH may be due to the lack of active K_ATP_ channels at the membrane.

### Dominant Mutation Carriers Developing Diabetes

A striking feature of dominant mutations is that dominant mutations carriers have the tendency to develop diabetes later in life (76). This was the case with five mothers of CHH patients who had developed gestational diabetes, and two of them experienced hypoglycemia during infancy. The proposed theory suggests that β-cell apoptosis, may be what is causing diabetes later in life (80). It has been claimed that as CHH is linked with β-cell depolarization, enhanced Ca^2+^ entry into the cell and overall increase in intracellular Ca^2+^ concentrations, then ultimately this increased energy demand required to transport the additional Ca^2+^ into the cell may be contributing to β-cell apoptosis. Therefore, the use of diazoxide may relieve the cell and delay the onset of β-cell apoptosis, and hinder the development of diabetes, as diazoxide reduces the influx of Ca^2+^.

However, there is controversy as to whether the dominant mutations predispose to diabetes later in life (79). Two large cohorts of dominant mutation carriers in different centers have shown opposing results (76, 78). Huopio et al. (76) saw that six out of eight mothers developed impaired glucose tolerance during pregnancy. However, Pinney et al. (78) had a larger group and did not see a positive trend toward diabetes in mutation carries. Only 3 out of 13 women had gestational diabetes. It has been argued that Sur1 KO mice or K_ir_6.2 KO mice do not demonstrate β-cell apoptosis or develop diabetes (78). And so there are clearly other pathways, which act to maintain insulin secretion independently of K_ATP_ channels. Therefore, the glucose intolerance in both dominant and recessive mutations is the result of defective K_ATP_ channels, and not a sign of developing diabetes in the future (78).

Kapoor et al. (81), however, showed that the main phenotype linked with dominant *ABCC8* and *KCNJ11* mutations is quite varied (81). It ranges from asymptomatic macrosomia to persistent HH in childhood. For adults who are mutation carriers, these mutations may be a potential cause of dominantly inherited early onset diabetes mellitus.

### Electrophysiology of K_ATP_ Channels in CHH

Studies involving the patch-clamp technique have shown that there are very few or almost no K_ATP_ channels on the surface on pancreatic β-cells from patients with diffuse, medically unresponsive CHH (71). The β-cells are depolarized, with variably high levels of intracellular Ca^2+^ in the presence of low glucose concentrations. The mutated K_ATP_ channels also exhibit no change in Ca^2+^ concentrations upon application of high glucose concentrations, tolbutamide, or diazoxide (82).

### Diazoxide Enhances Insulin Secretion via Unknown Mechanism

The collection of diseased pancreatic tissue from CHH patients who undergo surgery is a complicated process. The subsequent study of such tissue is made more problematic due to the challenging prospect of collecting healthy control tissue. Henquin et al. (82) collected suitable controls for comparison studies, and they have shown that diazoxide often caused an unexpected increase in insulin secretion (82). The results could not be considered as an artifact as pinacidil, which is also another K_ATP_ channel opener, was also reported to have increased the secretion of insulin. It is known that higher concentrations of diazoxide reduce the production of ATP by affecting the mitochondrial membrane potential, but this does not explain why more insulin is released from focal and diffuse tissue as less ATP should in fact decrease the secretion of insulin.

### Correction of Defective K_ATP_ Channels in Diseased Pancreatic Tissue

Powell and colleagues performed studies on pancreatic tissue obtained from CHH patients who underwent a pancreatectomy, and demonstrated the rescue of K_ATP_ channels (83). Inside-out patch-clamp recordings revealed that defective K_ATP_ channels had a normal response to diazoxide and ADP, when incubated at 25°C for 16 h. Whereas at 37°C, the defective K_ATP_ channels only showed between 0 and 10% activity compared to control cells. This work was based on the *ABCC7* gene, which encodes the cystic fibrosis transmembrane regulator (CFTR), where much research has been done to enhance the trafficking of CFTR to the cell membrane. This research may now be an invaluable tool to enhance the treatment of CHH, by rescuing the defective K_ATP_ channels (83).

## Metabolopathies

Metabolopathies is a term used to describe metabolic defects in seven genes (*GCK, SCHAD, GLUD1, SLC16A1, HNF1A, HNF4A*, and *UCP2*), which cause rare forms of CHH (84, 85). These genetic defects will be reviewed in this section.

### *GLUD1*: Glutamate Dehydrogenase-1

The *GLUD1* gene encodes for the glutamate dehydrogenase-1 (GDH) enzyme, which is expressed in the mitochondrial matrix. Amino acids are an essential source of fuel, which can be used as substitutes for glucose to generate ATP. The amino acid glutamate is interchangeably converted to α-ketoglutarate by the GDH enzyme, which is channeled into the Krebs cycle to generate ATP. The rising concentration of ATP triggers the depolarization of pancreatic β-cells through the closure of K_ATP_ channels, which culminates in the secretion of insulin.

The GDH enzyme is known to harbor two structural features, which are known to regulate its function. The first is the binding of leucine to the enzyme active site, and the second is an allosteric inhibitory binding site know to associate with nucleotides such as ATP and GTP (86). Dominantly inherited and *de novo* mutations in the *GLUD1* are known to cause CHH. The majority of mutations have been identified in exon 11 and 12 of the *GLUD1* gene (87); although, there are also reports of mutations in exon 6 and 7 (88). These activating mutations in the *GLUD1* gene desensitize the GDH enzyme to inhibition through ATP and GTP (89), causing re-current activation through the amino acid leucine. This leads to the oxidation of glutamate to α-ketoglutarate, which triggers an increase in the ratio of ATP/ADP, which subsequently enhances the secretion of insulin.

A striking feature of *GLUD1*–CHH patients is their sensitivity to protein, in particular to the amino acid leucine, as it has been noted that they can develop re-current symptomatic hypoglycemia after consuming a protein rich meal. Hence, *GLUD1*–CHH is also known as Hyperinsulinism-hyperammonemia syndrome (HI/HA). The HI/HA may due to renal ammoniagenesis (90). *GLUD1*–CHH patients present with high levels (two to three times the normal) of plasma ammonia; however, they do not develop the symptoms associated with hyperammonemia. These patients do, however, experience episodes of epilepsy, which suggests that this condition may directly affect the brain. The overall medical management of the disease relies on the use of the drug diazoxide, which is effective for *GLUD1*–CHH patients.

### *GCK*: Glucokinase (Hexokinase-4)

The enzyme, glucokinase, plays a fundamental role in “sensing” glucose in cells (91, 92). In pancreatic β-cells, the uptake of glucose occurs through an islet-specific glucose transporter, which is encoded by the *GLUT2* gene. Glucose enters the cell through glucose transporters embedded in cell membranes. Once inside the cell, a single molecule of glucose is chemically modified using the enzyme glucokinase, which phosphorylates it and converts it to glucose-6-phosphate. This in essence “traps” the glucose molecule inside the cell, and it can no longer exit the cell membrane. This enzyme provides a mechanism by which cells are able to store glucose and reduce the amount of glucose which is circulating in the bloodstream.

Dominant mutations in the *GCK* gene alter the enzyme’s affinity for glucose. The penultimate effect is on insulin secretion, which becomes unregulated due to the increasing concentration of glucose inside the β-cells. Thus dominant gain-of-function mutations in *GCK* cause CHH (93). A total of 15 mutations have been identified in this gene associated with CHH, which target various sites across the *GCK* gene (94). There are many other mutations associated with MODY2.

Patients presenting with *GCK*–CHH can have a variable phenotype to one another, as everything from the birth weight to the age of presentation to the severity of the disease is dissimilar (78). Although there are cases of patients responding to drugs such as diazoxide, others have required surgery to control the hypoglycemia.

### *HADH*: Short Chain L3-Hydroxyacyl-CoA Dehydrogenase

The oxidation of fatty acids and amino acids generates an alternative source of fuel that can be used to generate ATP. This is an essential process, which is highly used during fasting periods when the body is deprived of energy in form of dietary carbohydrates. The *HADH* gene is located on chromosome 4q22-26, and it codes for the enzyme Short chain L3-Hydroxyacyl-CoA dehydrogenase (SCHAD), which is involved in β-oxidation. This enzyme is expressed in many tissues, with high concentrations in the islet of Langerhans (95). However, its prime activity is specifically in the mitochondria, where it catalyzes the oxidation of different sized L3-hydroxacyl-CoA to their respective 3-ketoacyl-CoAs. The reaction requires the use of one NAD^+^ cofactor, and generates a single NADH as a product.

Autosomal recessive mutations in *HADH* are a rare cause of CHH (96), which leads to a reduction in the levels of the SCHAD enzyme. As a result, there is an accumulation of medium and short-chain fatty acids, which are not broken down properly, and therefore cannot enter the metabolic pathway leading to ATP production, which ultimately affects the K_ATP_ channel pathway. Other targets of *HADH* inactivity can effect G protein-coupled receptor GPR40 (97) as well play a role in the inhibition of carnitine palmitoyltransferase 1 (CPT-1) (98). *HADH* is known to be regulated by transcription factors such as *FOXA2*, which may play a role in the differentiation process of pancreatic β-cells in mice (99).

The clinical presentation of *HADH*–CHH is also a varied among patients with the disease. The patients are largely from consanguineous families, and the age of disease onset is unpredictable, with cases presenting at birth with severe CHH, as well other late onset with mild hypoglycemia. The first case of adult HH due to a *HADH* mutation has been reported, where the hypoglycemia became milder as the patient grew older (100).

Abnormal levels of hydroxybutyryl-acylcarnitine in blood and 3-hydroxyglutaric acid in urine are usually found in *HADH*–CHH patients. However, all cases have been known to be responsive to diazoxide, although patients may still suffer from a hypoglycemic episode after a protein-rich meal. Examples of *HADH* mutations include IVS6-2a > g. This is a splice site mutation generating, an enzyme which has ~90% reduction is activity in comparison to controls. The p.Met188Val mutant, however, had mild effects on enzyme activity, which originated in a patient with normal levels of acylcarnitines and organic acids.

It has been reported that some *HADH*–CHH patients are also leucine sensitive (101), however, unlike *GLUD1*–CHH patients, they do not present with hyperammonemia. The mechanism of leucine action in *HADH*–CHH patients is clearly different to *GLUD1*–CHH. A protein–protein interaction between SCHAD and GDH had been demonstrated in the liver (86). Furthermore, a *hadh* KO mouse model developed by Li et al. (86) had previously shown that leucine loading leads to a hyperinsulinemic response (86). With this in mind, the direct link between SCHAD and GDH in patient lymphoblast cells was first reported by Heslegrave et al. (101). It has been postulated that the regulation of the GDH enzyme may be affected by the ratio of active SCHAD to GDH in pancreatic β-cells. SCHAD may be an inhibitor of GDH, which suppresses the secretion of insulin via leucine stimulation (102). Loss of protein–protein interaction may cause a mild increase in activation of GDH (86). Patients with *HADH* mutations do have lower levels of SCHAD, although this is not the case in all patients, which may be the cause of the varying phenotypes seen in these patients. Further studies are needed to understand the mechanism of this interaction.

### *HNF4A*: Hepatocyte Nuclear Factor 4 Alpha

The *HNF4A* gene belongs to the nuclear hormone receptor superfamily. This gene harbors two distinct promoter regions known as P1 and P2. The transcription of the latter promoter is enhanced in the pancreatic β-cells, where it regulates key genes involved in glucose-stimulated insulin secretion (103, 104). Genes known to be regulated by *HNF4A* include glucose transporter-2, glycolytic enzymes, and enzymes involved in mitochondrial metabolism (104). A gene of significant interest is *KCNJ11*, which is also regulated by *HNF4A*. It has been hypothesized that *HNF4A* may down regulate the expression of the *KCNJ11* by up to 60%, although the precise mechanism is not known (105). The decreased expression was observed in mice using the conditional Cre-loxP-based inactivation system, which was used to delete the *HNF4A* gene. However conflicting evidence has shown by other researchers where the deletion of *HNF4A* had no effect on the activity or expression of *KCNJ11* (106, 107).

The peroxisome proliferator-activated receptor alpha (PPARα) is a transcription factor which may be an alternative target of *HNF4A* activity. PPARα is also involved in regulating the expression of enzymes involved in fatty acid oxidation, and during a fast PPARα, KO mice have been shown to develop HH (108). The association between the two transcription factors is based on the fact that there are reduced levels of PPARα in β-cells lacking *HNF4A* (105). Such a reduction of PPARα can lead to a reduction in the levels of β-oxidation, leading to the accumulation of lipids such as malonyl-CoA within the cytoplasm. This in turn can inhibit the enzyme CPT-1, which can raise the concentration of cytosolic long-chain acyl-CoA, terminating with the increased production of insulin.

Only a small fraction of patients with dominant inactivating *HNF4A* mutations actually go on to develop CHH (106, 109–111), and hence the penetrance of the disease is quite low. However, these patient’s do go on to develop maturity onset diabetes type 1 (MODY1), a form diabetes which is inherited in an autosomal dominant fashion (106). Another prominent feature in *HNF4A*–CHH patients is macrosomia. In comparison to family members, the *HNF4A*–CHH patients had a median increase in birth weight by 790 g in one cohort (106) and 751 g in another (109). This increase in birth weight is similar to patients with K_ATP_ channel mutations.

### *HNF1A*: Hepatocyte Nuclear Factor 1 Alpha

The *HNF1A* gene codes for a transcription factor, which is known to enhance the expression of liver specific genes, as well as genes expressed in the pancreas (112). Heterozygous, loss of function mutations in this gene are known to cause CHH. *HNF1A*–CHH patients present with milder fetal macrosomia, and are known to develop MODY3 as they age. The severity of the CHH is also less compared to patients with *HNF4A*–CHH. It is currently unknown as to why there is switch from CHH to MODY3 in these patients. *HNF1A*–CHH patients are responsive to drugs such as diazoxide. For the first time, Rozenkova and colleagues have demonstrated the functional impact of *HNF1A* mutations, which cause CHH. DNA-binding studies revealed that the p.Asn62Lysfs*93 and p.Leu254Gln *HNF1A* mutations reduced the transcriptional activity of the protein by 2.9 and 22%, respectively (113). The overall incidence of *HNF1A*–CHH is very low, and this could be due to the fact that some patients escape detection as their phenotype is very mild.

### *SLC16A1*: Solute Carrier Family 16, Member 1

The *SLC16A1* gene encodes for a transporter protein known as monocarboxylate transporter 1 (MCT1). Its function is to regulate the movement of lactate and pyruvate into the pancreatic β-cell. Under normal physiological conditions, lactate and pyruvate are present in low concentrations in the β-cell as they are potent insulin secretagogues. This transporter protein is not normally expressed (disinhibited) in pancreatic β-cells, but gain-of-function mutations in the promoter region of the gene enable its expression in β-cells, this allowing increased pyruvate into the β-cell and eventually leading to CHH. A unique feature about these patients is that the onset of the disease is exercise induced. Hypoglycemia was observed in these patients after around 30–45 min of strenuous exercise. These features were prominent in Finnish cohort of 13 patients (114). In these patients, lactate is produced during anaerobic exercise, and this is fed into the Krebs cycle together with pyruvate, which generates ATP and triggers insulin release through the K_ATP_ channel pathway. Most patients are responsive to medical management using diazoxide, although this is ineffective after anaerobic exercise, hence strenuous exercise is not tolerated by these patients (115).

### *UCP2*: Uncoupling Protein 2

The *UCP2* gene codes for a mitochondrial carrier protein largely expressed in both pancreatic α- and β-cells. The structure of the UCP2 protein entails six transmembrane domains (116) and one nucleotide-binding domain. It has been hypothesized that this gene provides protection against oxidative stress during fatty acid metabolism. *In vivo* studies observing UCP2 KO mice have shown that these mice develop hyperinsulinism, which suggests that *UCP2* is involved in the regulation of insulin secretion. In animal models, it has also been shown that the overexpression of *ucp2* can cause diabetes. Hence there is a clear involvement of *UCP2* in glucose physiology. It has been hypothesized that the UCP2 protein may be involved in regulating the leak of protons across the inner mitochondrial membrane (117). This would lead to the uncoupling of mitochondrial oxidative metabolism from ATP synthesis, which ultimately reduces the ATP yield for substrate oxidation.

González-Barroso et al. (118) described the first two cases of CHH due to mutations in the *UCP2* gene (118). The age of presentation in these two patients was later around ~8 months. Insulinoma and yeast cells have been used to further understand the link between *UCP2* and HH. Studies have revealed that mutations in *UCP2* have a loss-of-function effect on the protein, which enhances insulin secretion, whereas the wild type (WT) *UCP2* protein inhibits insulin secretion (118). Further studies are warranted in order to establish a clear mechanism of *UCP2*-induced HH.

### Insulin Receptor Gene Mutation

A report of a novel heterozygous mutation in the insulin receptor gene has been causative of hypoglycemia in patients with severe HH (119). The autosomal dominant change in the insulin receptor gene (p.Arg1174Gln) has been described to cause postprandial hyperinsulinemic hypoglycemia (PPHH) (119). PPHH occurs after a few hours of eating a meal. PPHH is secondary to the unregulated insulin release due to the meal ingested.

The hyperinsulinism seen in these patients appear to be due to a reduction in the clearance of insulin rather than the increased release of insulin, since C-peptide concentrations are within normal ranges.

## Non-Genetic Causes of HH

### Postprandial Hyperinsulinemic Hypoglycemia

Other non-genetic causes of PPHH also exist (Table 1):

### Dumping Syndrome

Dumping syndrome is an example of PPHH, which occurs after Nissen’s fundoplication (120). Ingestion of carbohydrate-rich foods is absorbed into the small intestine, which in turn cause increased glucose absorption, hyperglycemia, rapid release of insulin, and overall results in hypoglycemia. The molecular mechanisms of this is thought to be due to abnormally exaggerated levels of glucagon-like peptide-1 (GLP-1), which is known to contribute to the HH (121).

Where dumping syndrome is suspected, a mixed meal or oral glucose tolerance test (OGTT) is performed. A current diagnostic criteria for dumping syndrome is a decrease of more than 6 mmol/L between peak and nadir blood glucose levels (122).

### Insulin Autoimmune Syndrome

Insulin autoimmune syndrome is also known as Hirata disease, which is a rare cause of hypoglycemia characterized by high levels of endogenous insulin autoantibodies. This condition has the absence of pathological islets and is in the absence of exposure to exogenous insulin (123). Insulin autoimmunity affects mainly adults of 40 years and over.

In this condition, post meal or after a glucose load, the raised blood glucose levels cause insulin secretion but hyperglycemia occurs. This is due to the insulin antibodies binding to insulin and reducing the bioavailability to its receptor and this in turn leads to a reduction in its action. After a few hours, the blood glucose levels decrease with corresponding insulin concentrations, and the bound insulin is released from the antibodies, resulting in the hypoglycemia (124).

### Bariatric Surgery

Obese individuals with comorbidities such a type-2 diabetes mellitus are causing a rise in invasive gastric bypass procedures (125). Prior to weight-loss, these patients have shown a resolution of their hyperglycemia. The theory for this mechanism is that the food bypasses the upper gastrointestinal tract and is passed along to the lower gut, here the insulinotropic hormone; GLP-1 is hyperstimulated (126). However, a serious side effect appears to be an increase in gastric bypass patients presenting with PPHH and nesidioblastosis (127).

### Non-Insulinoma Pancreatogenous Hypoglycemia Syndrome

Postprandial neuroglycopenia characterizes the non-insulinoma pancreatogenous hypoglycemia syndrome (NIPHS) (128). The genetic cause of NIPHS remains to be found, these patients are negative for prolonged fasts and show islet hypertrophy and evidence of nesidioblastosis in the absence of insulinoma. However, studies are yet to identify if the increased β-cell proliferation production as well as pancreatic differentiation factors are contributing agents (129). In the absence of K_ATP_ channel mutations, some of these patients are surprisingly responsive to diazoxide (130), while the unresponsive patients require a partial pancreatectomy.

### Insulinoma

In adults, insulinomas are the most common cause of endogenous HH with an incidence of four per million (131). Predominantly of benign origin, these pancreatic tumors secrete insulin and are usually small and <2 mm in diameter. Initially, hypoglycemia was thought to show only either on fasting or post-exercise; however, it has also presented post meal (132). In suspected cases, current guidelines recommend the measurement of plasma glucose, insulin, C-peptide, proinsulin, β-hydroxybutyrate, insulin antibodies, and circulating oral hypoglycemic agents during the hypoglycemic episode (133).

## Conclusion

Hyperinsulinemic hypoglycemia is characterized by the dysregulation of insulin secretion from pancreatic β-cells. The most severe forms of congenital hyperinsulinemic hypoglycemia are due to molecular defects in the genes *ABCC8* and *KCNJ11*. Milder forms of hyperinsulinemic hypoglycemia may occur due to molecular defects in several other genes, which play a key role in the regulation of insulin secretion. Currently genetic abnormalities are only found in about 50% of patients presenting with hyperinsulinemic hypoglycemia. Understanding the molecular mechanisms of dysregulated insulin secretion in those patients with no genetic etiology will provide novel insights into pancreatic β-cell function.

## Author Contributions

In this review article, AN wrote the following parts: (1) introduction (wrote the majority of this section, except for the first three paragraphs), (2) K_ATP_ channels and insulin secretion (wrote the entire section), (3) metabolopathies (wrote the entire section, except the part on “insulin receptor gene mutation”), and (4) figures (generated all the three figures). SR wrote the following parts: (1) introduction (wrote the starting paragraphs), (2) histological characterisation of CHH, (3) metabolopathies (section listed below): (a) insulin receptor gene mutation, (4) non-genetic causes of HH (wrote the entire section), (5) conclusion, and (6) tables (generated Table 1). KH wrote the following parts: (1) abstract, (2) read through the entire review article and corrected for grammar, and (3) also contributed to the style and structure of the article.

## Conflict of Interest Statement

The authors declare that the research was conducted in the absence of any commercial or financial relationships that could be construed as a potential conflict of interest.

## References

[1] Güemes M Rahman SA Hussain K What is a normal blood glucose? Arch Dis Child (2015). 10.1136/archdischild-2015-308336 26369574

[2] Aynsley-Green A Hussain K Hall J Saudubray JM Nihoul-Fékété C De Lonlay-Debeney P Practical management of hyperinsulinism in infancy. Arch Dis Child Fetal Neonatal Ed (2000) 82(2):F98–107. 10.1136/fn.82.2.F98 10685981 PMC1721064

[3] Hussain K Bryan J Christesen HT Brusgaard K Aguilar-Bryan L. Serum glucagon counterregulatory hormonal response to hypoglycemia is blunted in congenital hyperinsulinism. Diabetes (2005) 54(10):2946–51. 10.2337/diabetes.54.10.2946 16186397

[4] Hussain K Hindmarsh P Aynsley-Green A. Neonates with symptomatic hyperinsulinemic hypoglycemia generate inappropriately low serum cortisol counterregulatory hormonal responses. J Clin Endocrinol Metab (2003) 88(9):4342–7. 10.1210/jc.2003-030135 12970308

[5] Meissner T Wendel U Burgard P Schaetzle S Mayatepek E. Long-term follow-up of 114 patients with congenital hyperinsulinism. Eur J Endocrinol (2003) 149(1):43–51. 10.1530/eje.0.1490043 12824865

[6] Kapoor RR Flanagan SE James C Shield J Ellard S Hussain K. Hyperinsulinaemic hypoglycaemia. Arch Dis Child (2009) 94(6):450–7. 10.1136/adc.2008.148171 19193661

[7] Thomas P Ye Y Lightner E. Mutation of the pancreatic islet inward rectifier Kir6.2 also leads to familial persistent hyperinsulinemic hypoglycemia of infancy. Hum Mol Genet (1996) 5(11):1809–12. 10.1093/hmg/5.11.1809 8923010

[8] Thomas PM Cote GJ Wohllk N Haddad B Mathew PM Rabl W Mutations in the sulfonylurea receptor gene in familial persistent hyperinsulinemic hypoglycemia of infancy. Science (1995) 268(5209):426–9. 10.1126/science.7716548 7716548

[9] Dunne MJ Cosgrove KE Shepherd RM Aynsley-Green A Lindley KJ. Hyperinsulinism in infancy: from basic science to clinical disease. Physiol Rev (2004) 84(1):239–75. 10.1152/physrev.00022.2003 14715916

[10] Phillips R Quake SR The biological frontier of physics. Phys Today (2006) 59(5):38–43. 10.1063/1.2216960

[11] Dean M Annilo T Evolution of the ATP-binding cassette (ABC) transporter superfamily in verterbrates. Annu Rev Genomics Hum Genet (2005) 6(1):123–42. 10.1146/annurev.genom.6.080604.162122 16124856

[12] Rees DC Johnson E Lewinson O. ABC transporters: the power to change. Nat Rev Mol Cell Biol (2009) 10(3):218–27. 10.1038/nrm2646 19234479 PMC2830722

[13] Walker JE Saraste M Runswick MJ Gay NJ. Distantly related sequences in the alpha- and beta-subunits of ATP synthase, myosin, kinases and other ATP-requiring enzymes and a common nucleotide binding fold. EMBO J (1982) 1(8):945–51. 6329717 10.1002/j.1460-2075.1982.tb01276.x PMC553140

[14] Inagaki N Gonoi T Clement JP Namba N Inazawa J Gonzalez G Reconstitution of IKATP: an inward rectifier subunit plus the sulfonylurea receptor. Science (1995) 270(5239):1166–70. 10.1126/science.270.5239.1166 7502040

[15] Nestorowicz A Wilson BA Schoor KP Inoue H Glaser B Landau H Mutations in the sulfonylurea receptor gene are associated with familial hyperinsulinism in Ashkenazi Jews. Hum Mol Genet (1996) 5(11):1813–22. 10.1093/hmg/5.11.1813 8923011

[16] Aguilar-Bryan L Nelson DA Vu QA Humphrey MB Boyd AE. Photoaffinity labeling and partial purification of the beta cell sulfonylurea receptor using a novel, biologically active glyburide analog. J Biol Chem (1990) 265(14):8218–24. 2110566

[17] Aguilar-Bryan L Clement JP Gonzalez G Kunjilwar K Babenko A Bryan J. Toward understanding the assembly and structure of KATP channels. Physiol Rev (1998) 78(1):227–45. 9457174 10.1152/physrev.1998.78.1.227

[18] Gribble FM Reimann F. Sulphonylurea action revisited: the post-cloning era. Diabetologia (2003) 46(7):875–91. 10.1007/s00125-003-1143-3 12819907

[19] Ashcroft FM Gribble FM. New windows on the mechanism of action of K(ATP) channel openers. Trends Pharmacol Sci (2000) 21(11):439–45. 10.1016/S0165-6147(00)01563-7 11121575

[20] Gribble FM Tucker SJ Ashcroft FM. The essential role of the Walker A motifs of SUR1 in K-ATP channel activation by Mg-ADP and diazoxide. EMBO J (1997) 16(6):1145–52. 10.1093/emboj/16.6.1145 9135131 PMC1169713

[21] Schlichting I Almo SC Rapp G Wilson K Petratos K Lentfer A Time-resolved X-ray crystallographic study of the conformational change in Ha-Ras p21 protein on GTP hydrolysis. Nature (1990) 345(6273):309–15. 10.1038/345309a0 2111463

[22] Dean M Allikmets R. Complete characterization of the human ABC gene family. J Bioenerg Biomembr (2001) 33(6):475–9. 10.1023/a:1012823120935 11804189

[23] Matsuo M Kioka N Amachi T Ueda K. ATP binding properties of the nucleotide-binding folds of SUR1. J Biol Chem (1999) 274:37479–82. 10.1074/jbc.274.52.37479 10601323

[24] Hough E Mair L Mackenzie W Sivaprasadarao A. Expression, purification, and evidence for the interaction of the two nucleotide-binding folds of the sulphonylurea receptor. Biochem Biophys Res Commun (2002) 294(1):191–7. 10.1016/S0006-291X(02)00454-0 12054762

[25] Mikhailov MV Ashcroft SJ. Interactions of the sulfonylurea receptor 1 subunit in the molecular assembly of beta-cell K(ATP) channels. J Biol Chem (2000) 275(5):3360–4. 10.1074/jbc.275.5.3360 10652326

[26] Doupnik CA Davidson N Lester HA. The inward rectifier potassium channel family. Curr Opin Neurobiol (1995) 5(3):268–77. 10.1016/0959-4388(95)80038-7 7580148

[27] Ruppersberg JP Intracellular regulation of inward rectifier K+ channels. Pflügers Arch (2000) 441(1):1–11. 10.1007/s004240000380 11205046

[28] Krapivinsky G Medina I Eng L Krapivinsky L Yang Y Clapham DE. A novel inward rectifier K+ channel with unique pore properties. Neuron (1998) 20(5):995–1005. 10.1016/S0896-6273(00)80480-8 9620703

[29] Sempoux C Guiot Y Jaubert F Rahier J. Focal and diffuse forms of congenital hyperinsulinism: the keys for differential diagnosis. Endocr Pathol (2004) 15(3):241–6. 10.1385/ep:15:3:241 15640550

[30] Goossens A Gepts W Saudubray JM Bonnefont JP Nihoul F Heitz PU Diffuse and focal nesidioblastosis. A clinicopathological study of 24 patients with persistent neonatal hyperinsulinemic hypoglycemia. Am J Surg Pathol (1989) 13(9):766–75. 10.1097/00000478-198909000-00006 2669541

[31] Rahier J Falt K Muntefering H Becker K Gepts W Falkmer S. The basic structural lesion of persistent neonatal hypoglycaemia with hyperinsulinism: deficiency of pancreatic D cells or hyperactivity of B cells? Diabetologia (1984) 26(4):282–9. 10.1007/BF00283651 6376236

[32] Rahier J Guiot Y Sempoux C Persistent hyperinsulinaemic hypoglycaemia of infancy: a heterogeneous syndrome unrelated to nesidioblastosis. Arch Dis Child Fetal Neonatal Ed (2000) 82(2):F108–12. 10.1136/fn.82.2.F108 10685982 PMC1721069

[33] Sempoux C Guiot Y Lefevre A Nihoul-Fekete C Jaubert F Saudubray JM Neonatal hyperinsulinemic hypoglycemia: heterogeneity of the syndrome and keys for differential diagnosis. J Clin Endocrinol Metab (1998) 83(5):1455–61. 10.1210/jc.83.5.1455 9589638

[34] Sempoux C Capito C Bellanné-Chantelot C Verkarre V de Lonlay P Aigrain Y Morphological mosaicism of the pancreatic islets: a novel anatomopathological form of persistent hyperinsulinemic hypoglycemia of infancy. J Clin Endocrinol Metabol (2011) 96(12):3785–93. 10.1210/jc.2010-3032 21956412

[35] Damaj L le Lorch M Verkarre V Werl C Hubert L Nihoul-Fekete C Chromosome 11p15 paternal isodisomy in focal forms of neonatal hyperinsulinism. J Clin Endocrinol Metab (2008) 93(12):4941–7. 10.1210/jc.2008-0673 18796520

[36] Rahier J Sempoux C Fournet JC Poggi§ F Brunelle F Nihoul-Fekete C Partial or near-total pancreatectomy for persistent neonatal hyperinsulinaemic hypoglycaemia: the pathologist’s role. Histopathology (1998) 32(1):15–9. 10.1046/j.1365-2559.1998.00326.x 9522211

[37] Otonkoski T Näntö-Salonen K Seppänen M Veijola R Huopio H Hussain K Noninvasive diagnosis of focal hyperinsulinism of infancy with [18F]-DOPA positron emission tomography. Diabetes (2006) 55(1):13–8. 10.2337/diabetes.55.01.06.db05-1128 16380471

[38] Hussain K Flanagan SE Smith VV Ashworth M Day M Pierro A An ABCC8 gene mutation and mosaic uniparental isodisomy resulting in atypical diffuse congenital hyperinsulinism. Diabetes (2008) 57(1):259–63. 10.2337/db07-0998 17942822

[39] Henquin J-C Sempoux C Marchandise J Godecharles S Guiot Y Nenquin M Congenital hyperinsulinism caused by hexokinase I expression or glucokinase-activating mutation in a subset of β-cells. Diabetes (2013) 62(5):1689–96. 10.2337/db12-1414 23274908 PMC3636634

[40] Shi Y Avatapalle HB Skae MS Padidela R Newbould M Rigby L Increased plasma incretin concentrations identifies a subset of patients with persistent congenital hyperinsulinism without KATP channel gene defects. J Pediatr (2015) 166(1):191–4. 10.1016/j.jpeds.2014.09.019 25444530

[41] Noma A ATP-regulated K+ channels in cardiac muscle. Nature (1983) 305(5930):147–8. 10.1038/305147a0 6310409

[42] Spruce AE Standen NB Stanfield PR. Voltage-dependent ATP-sensitive potassium channels of skeletal muscle membrane. Nature (1985) 316(6030):736–8. 10.1038/316736a0 2412127

[43] Cook DL Hales CN. Intracellular ATP directly blocks K+ channels in pancreatic B-cells. Nature (1984) 311(5983):271–3. 10.1038/311271a0 6090930

[44] Standen N Quayle J Davies N Brayden J Huang Y Nelson M. Hyperpolarizing vasodilators activate ATP-sensitive K+ channels in arterial smooth muscle. Science (1989) 245(4914):177–80. 10.1126/science.2501869 2501869

[45] Ashcroft FM Adenosine 5’-triphosphate-sensitive potassium channels. Annu Rev Neurosci (1988) 11(1):97–118. 10.1146/annurev.ne.11.030188.000525 2452599

[46] Salari S Ghasemi M Fahanik-Babaei J Saghiri R Sauve R Eliassi A. Evidence for a K_ATP_ channel in rough endoplasmic reticulum (rerK_ATP_ channel) of rat hepatocytes. PLoS One (2015) 10(5):e0125798. 10.1371/journal.pone.0125798 25950903 PMC4423865

[47] Rorsman P Trube G Glucose dependent K+ channels in pancreatic beta-cells are regulated by intracellular ATP. Pflugers Arch (1985) 405(4):305–9. 10.1007/BF00595682 2417189

[48] Henquin JC. Tolbutamide stimulation and inhibition of insulin release: studies of the underlying ionic mechanisms in isolated rat islets. Diabetologia (1980) 18(2):151–60. 10.1007/BF00290493 6988275

[49] Henquin JC. The potassium permeability of pancreatic islet cells: mechanisms of control and influence on insulin release. Horm Metab Res (1980) 10:66–73. 7005065

[50] Meissner HP. Electrical characteristics of the beta-cells in pancreatic islets. J Physiol (Paris) (1976) 72(2):757–67. 792422

[51] Trube G Rorsman P Ohno-Shosaku T. Opposite effects of tolbutamide and diazoxide on the ATP-dependent K+ channel in mouse pancreatic beta-cells. Pflugers Arch (1986) 407(5):493–9. 10.1007/BF00657506 2431383

[52] Ashcroft FM Kakei M. ATP-sensitive K+ channels in rat pancreatic beta-cells: modulation by ATP and Mg2+ ions. J Physiol (1989) 416:349–67. 10.1113/jphysiol.1989.sp017765 2691645 PMC1189219

[53] Rajan AS Aguilar-Bryan L Nelson DA Nichols CG Wechsler SW Lechago J Sulfonylurea receptors and ATP-sensitive K+ channels in clonal pancreatic alpha cells. Evidence for two high affinity sulfonylurea receptors. J Biol Chem (1993) 268(20):15221–8. 8325894

[54] Shimono D Fujimoto S Mukai E Takehiro M Nabe K Radu RG ATP enhances exocytosis of insulin secretory granules in pancreatic islets under Ca2+ depleted condition. Diabetes Res Clin Pract (2005) 69(3):216–23. 10.1016/j.diabres.2005.01.010 16098917

[55] Shyng S-L Nichols CG. Membrane phospholipid control of nucleotide sensitivity of KATP channels. Science (1998) 282(5391):1138–41. 10.1126/science.282.5391.1138 9804554

[56] Suh B-C Hille B. PIP2 is a necessary cofactor for ion channel function: how and why? Annu Rev Biophys (2008) 37(1):175–95. 10.1146/annurev.biophys.37.032807.125859 18573078 PMC2692585

[57] Ribalet B John SA Xie L-H Weiss JN. ATP-sensitive K+ channels: regulation of bursting by the sulphonylurea receptor, PIP2 and regions of Kir6.2. J Physiol (2006) 571(2):303–17. 10.1113/jphysiol.2005.100719 16373383 PMC1796795

[58] Proks P Capener CE Jones P Ashcroft FM. Mutations within the P-loop of Kir6.2 modulate the intraburst kinetics of the ATP-sensitive potassium channel. J Gen Physiol (2001) 118:341–53. 10.1085/jgp.118.4.341 11585848 PMC2233698

[59] Nichols CG. KATP channels as molecular sensors of cellular metabolism. Nature (2006) 440(7083):470–6. 10.1038/nature04711 16554807

[60] Enkvetchakul D Loussouarn G Makhina E Shyng SL Nichols CG The kinetic and physical basis of KATP channel gating: toward a unified molecular understanding. Biophys J (2000) 78:2334–48. 10.1016/S0006-3495(00)76779-8 10777731 PMC1300824

[61] Markworth E Schwanstecher C Schwanstecher M. ATP4- mediates closure of pancreatic beta-cell ATP-sensitive potassium channels by interaction with 1 of 4 identical sites. Diabetes (2000) 49:1413–8. 10.2337/diabetes.49.9.1413 10969823

[62] Gillis KD Gee WM Hammoud A McDaniel ML Falke LC Misler S. Effects of sulfonamides on a metabolite-regulated ATPi-sensitive K+ channel in rat pancreatic B-cells. Am J Physiol (1989) 257(6 Pt 1):C1119–27. 2514595 10.1152/ajpcell.1989.257.6.C1119

[63] Nichols CG Lederer WJ Cannell MB. ATP dependence of KATP channel kinetics in isolated membrane patches from rat ventricle. Biophys J (1991) 60(5):1164–77. 10.1016/S0006-3495(91)82152-X 1760506 PMC1260172

[64] Trapp S Proks P Tucker SJ Ashcroft FM. Molecular analysis of ATP-sensitive K channel gating and implications for channel inhibition by ATP. J Gen Physiol (1998) 112(3):333–49. 10.1085/jgp.112.3.333 9725893 PMC2229413

[65] Li L Geng X Drain P. Open state destabilization by atp occupancy is mechanism speeding burst exit underlying KATP channel inhibition by ATP. J Gen Physiol (2002) 119(1):105–16. 10.1085/jgp.119.1.105 11773242 PMC2233857

[66] Craig TJ Ashcroft FM Proks P How ATP Inhibits the open KATP channel. J Gen Physiol (2008) 132(1):131–44. 10.1085/jgp.200709874 18591420 PMC2442177

[67] Ebel H Günther T. Magnesium metabolism: a review. J Clin Chem Clin Biochem (1980) 18(5):257–70. 7000968 10.1515/cclm.1980.18.5.257

[68] Aguilar-Bryan L Bryan J. Molecular biology of adenosine triphosphate-sensitive potassium channels. Endocr Rev (1999) 20(2):101–35. 10.1210/er.20.2.101 10204114

[69] Shyng S Nichols CG. Octameric stoichiometry of the KATP channel complex. J Gen Physiol (1997) 110:655–64. 10.1085/jgp.110.6.655 9382894 PMC2229396

[70] Tucker SJ Gribble FM Zhao C Trapp S Ashcroft FM. Truncation of Kir6.2 produces ATP-sensitive K+ channels in the absence of the sulphonylurea receptor. Nature (1997) 387(6629):179–83. 10.1038/387179a0 9144288

[71] Charlotte Kane RMS Squires PE Johnson PR James RF Milla PJ Aynsley-Green A Loss of functional KATP channels in pancreatic beta−cells causes persistent hyperinsulinemic hypoglycemia of infancy. Nat Med (1996) 2:1344–7. 10.1038/nm1296-1344 8946833

[72] Zerangue N Schwappach B Jan YN Jan LY A new ER trafficking signal regulates the subunit stoichiometry of plasma membrane KATP channels. Neuron (1999) 22(3):537–48. 10.1016/S0896-6273(00)80708-4 10197533

[73] Neagoe I Schwappach B Pas de deux in groups of four – the biogenesis of KATP channels. J Mol Cell Cardiol (2005) 38:887–94. 10.1016/j.yjmcc.2004.11.023 15910873

[74] Shyng SL Ferrigni T Shepard JB Nestorowicz A Glaser B Permutt MA Functional analyses of novel mutations in the sulfonylurea receptor 1 associated with persistent hyperinsulinemic hypoglycemia of infancy. Diabetes (1998) 47(7):1145–51. 10.2337/diabetes.47.7.1145 9648840

[75] Matsuo M Trapp S Tanizawa Y Kioka N Amachi T Oka Y Functional analysis of a mutant sulfonylurea receptor, SUR1-R1420C, that is responsible for persistent hyperinsulinemic hypoglycemia of infancy. J Biol Chem (2000) 275(52):41184–91. 10.1074/jbc.M006503200 10993895

[76] Huopio H Reimann F Ashfield R Komulainen J Lenko H-L Rahier J Dominantly inherited hyperinsulinism caused by a mutation in the sulfonylurea receptor type 1. J Clin Invest (2000) 106(7):897–906. 10.1172/JCI9804 11018078 PMC381424

[77] MacMullen CM Zhou Q Snider KE Tewson PH Becker SA Aziz AR Diazoxide-unresponsive congenital hyperinsulinism in children with dominant mutations of the β-cell sulfonylurea receptor SUR1. Diabetes (2011) 60(6):1797–804. 10.2337/db10-1631 21536946 PMC3114386

[78] Pinney SE MacMullen C Becker S Lin Y-W Hanna C Thornton P Clinical characteristics and biochemical mechanisms of congenital hyperinsulinism associated with dominant KATP channel mutations. J Clin Invest (2008) 118(8):2877–86. 10.1172/JCI35414 18596924 PMC2441858

[79] Thornton PS MacMullen C Ganguly A Ruchelli E Steinkrauss L Crane A Clinical and molecular characterization of a dominant form of congenital hyperinsulinism caused by a mutation in the high-affinity sulfonylurea receptor. Diabetes (2003) 52(9):2403–10. 10.2337/diabetes.52.9.2403 12941782

[80] Glaser B Ryan F Donath M Landau H Stanley CA Baker L Hyperinsulinism caused by paternal-specific inheritance of a recessive mutation in the sulfonylurea-receptor gene. Diabetes (1999) 48(8):1652–7. 10.2337/diabetes.48.8.1652 10426386

[81] Kapoor RR Flanagan SE James CT McKiernan J Thomas AM Harmer SC Hyperinsulinaemic hypoglycaemia and diabetes mellitus due to dominant ABCC8/KCNJ11 mutations. Diabetologia (2011) 54(10):2575–83. 10.1007/s00125-011-2207-4 21674179 PMC3168751

[82] Henquin JC Nenquin M Sempoux C Guiot Y Bellanné-Chantelot C Otonkoski T In vitro insulin secretion by pancreatic tissue from infants with diazoxide-resistant congenital hyperinsulinism deviates from model predictions. J Clin Invest (2011) 121(10):3932–42. 10.1172/JCI58400 21968111 PMC3195476

[83] Powell PD Bellanné-Chantelot C Flanagan SE Ellard S Rooman R Hussain K In vitro recovery of ATP-sensitive potassium channels in β-cells from patients with congenital hyperinsulinism of infancy. Diabetes (2011) 60(4):1223–8. 10.2337/db10-1443 21411514 PMC3064095

[84] Kapoor RR Flanagan SE Arya VB Shield JP Ellard S Hussain K. Clinical and molecular characterisation of 300 patients with congenital hyperinsulinism. Eur J Endocrinol (2013) 168(4):557–64. 10.1530/eje-12-0673 23345197 PMC3599069

[85] Snider KE Becker S Boyajian L Shyng S-L MacMullen C Hughes N Genotype and phenotype correlations in 417 children with congenital hyperinsulinism. J Clin Endocrinol Metabol (2013) 98(2):E355–63. 10.1210/jc.2012-2169 23275527 PMC3565119

[86] Li C Najafi H Daikhin Y Nissim IB Collins HW Yudkoff M Regulation of leucine-stimulated insulin secretion and glutamine metabolism in isolated rat islets. J Biol Chem (2003) 278(5):2853–8. 10.1074/jbc.M210577200 12444083

[87] Stanley C Fang J Kutyna K Hsu B Ming J Glaser B Molecular basis and characterization of the hyperinsulinism/hyperammonemia syndrome: predominance of mutations in exons 11 and 12 of the glutamate dehydrogenase gene. Diabetes (2000) 49:667–73. 10.2337/diabetes.49.4.667 10871207

[88] Santer R Kinner M Passarge M Superti-Furga A Mayatepek E Meissner T Novel missense mutations outside the allosteric domain of glutamate dehydrogenase are prevalent in European patients with the congenital hyperinsulinism-hyperammonemia syndrome. Hum Genet (2001) 108(1):66–71. 10.1007/s004390000432 11214910

[89] Stanley CA Lieu YK Hsu BYL Burlina AB Greenberg CR Hopwood NJ Hyperinsulinism and hyperammonemia in infants with regulatory mutations of the glutamate dehydrogenase gene. N Engl J Med (1998) 338(19):1352–7. 10.1056/NEJM199805073381904 9571255

[90] Treberg J Clow K Greene K Brosnan M Brosnan J. Systemic activation of glutamate dehydrogenase increases renal ammoniagenesis: implications for the hyperinsulinism/hyperammonemia syndrome. Am J Physiol Endocrinol Metab (2010) 298:E1219–25. 10.1152/ajpendo.00028.2010 20332361

[91] Johnson JH Newgard CB Milburn JL Lodish HF Thorens B. The high Km glucose transporter of islets of Langerhans is functionally similar to the low affinity transporter of liver and has an identical primary sequence. J Biol Chem (1990) 265(12):6548–51. 2182619

[92] Thorens B GLUT2, glucose sensing and glucose homeostasis. Diabetologia (2014) 58(2):221–32. 10.1007/s00125-014-3451-1 25421524

[93] Glaser B Kesavan P Heyman M Davis E Cuesta A Buchs A Familial hyperinsulinism caused by an activating glucokinase mutation. N Engl J Med (1998) 338(4):226–30. 10.1056/NEJM199801223380404 9435328

[94] Matschinsky FM Regulation of pancreatic β-cell glucokinase: from basics to therapeutics. Diabetes (2002) 51(Suppl 3):S394–404. 10.2337/diabetes.51.2007.S394 12475782

[95] Agren A Borg K Brolin S Carlman J Lundqvist G. Hydroxyacyl CoA dehydrogenase, an enzyme important in fat metabolism in different cell types in the islets of Langerhans. Diabete Metab (1977) 3:169–72. 334589

[96] Clayton P Eaton S Aynsley-Green A Edginton M Hussain K Krywawych S Hyperinsulinism in short-chain L-3-hydroxyacyl-CoA dehydrogenase deficiency reveals the importance of beta-oxidation in insulin secretion. J Clin Invest (2001) 108:457–65. 10.1172/JCI200111294 11489939 PMC209352

[97] Itoh Y Kawamata Y Harada M Kobayashi M Fujii R Fukusumi S Free fatty acids regulate insulin secretion from pancreatic [beta] cells through GPR40. Nature (2003) 422(6928):173–6. 10.1038/nature01478 12629551

[98] Eaton S Chatziandreou I Krywawych S Pen S Clayton PT Hussain K Short-chain 3-hydroxyacyl-CoA dehydrogenase deficiency associated with hyperinsulinism: a novel glucose–fatty acid cycle? Biochem Soc Trans (2003) 31(6):1137–9. 10.1042/bst0311137 14641012

[99] Lantz KA Vatamaniuk MZ Brestelli JE Friedman JR Matschinsky FM Kaestner KH. Foxa2 regulates multiple pathways of insulin secretion. J Clin Investig (2004) 114(4):512–20. 10.1172/JCI200421149 15314688 PMC503770

[100] Babiker O Flanagan SE Ellard S Girim HA Hussain K Senniappan S. Protein-induced hyperinsulinaemic hypoglycaemia due to a homozygous HADH mutation in three siblings of a Saudi family. J Pediatr Endocrinol Metab (2015) 28(9–10):1073–7. 10.1515/jpem-2015-0033 25915078

[101] Heslegrave A Kapoor R Eaton S Chadefaux B Akcay T Simsek E Leucine-sensitive hyperinsulinaemic hypoglycaemia in patients with loss of function mutations in 3-Hydroxyacyl-CoA Dehydrogenase. Orphanet J Rare Dis (2012) 7(1):25. 10.1186/1750-1172-7-25 22583614 PMC3433310

[102] Filling C Keller B Hirschberg D Marschall H Jornvall H Bennett M Role of short-chain hydroxyacyl CoA dehydrogenases in SCHAD deficiency. Biochem Biophys Res Commun (2008) 368:6–11. 10.1016/j.bbrc.2007.10.188 18036338

[103] Odom DT Zizlsperger N Gordon DB Bell GW Rinaldi NJ Murray HL Control of pancreas and liver gene expression by HNF transcription factors. Science (2004) 303(5662):1378–81. 10.1126/science.1089769 14988562 PMC3012624

[104] Wang H Maechler P Antinozzi PA Hagenfeldt KA Wollheim CB Hepatocyte nuclear factor 4α regulates the expression of pancreatic β-cell genes implicated in glucose metabolism and nutrient-induced insulin secretion. J Biol Chem (2000) 275(46):35953–9. 10.1074/jbc.M006612200 10967120

[105] Gupta RK Vatamaniuk MZ Lee CS Flaschen RC Fulmer JT Matschinsky FM The MODY1 gene HNF-4alpha regulates selected genes involved in insulin secretion. J Clin Invest (2005) 115(4):1006–15. 10.1172/JCI200522365 15761495 PMC1059446

[106] Pearson ER Boj SF Steele AM Barrett T Stals K Shield JP Macrosomia and hyperinsulinaemic hypoglycaemia in patients with heterozygous mutations in the *HNF4A* gene. PLoS Med (2007) 4(4):e118. 10.1371/journal.pmed.0040118 17407387 PMC1845156

[107] Miura A Yamagata K Kakei M Hatakeyama H Takahashi N Fukui K Hepatocyte nuclear factor-4α is essential for glucose-stimulated insulin secretion by pancreatic β-cells. J Biol Chem (2006) 281(8):5246–57. 10.1074/jbc.M507496200 16377800

[108] Gremlich S Nolan C Roduit R Burcelin R Peyot ML Delghingaro-Augusto V Pancreatic islet adaptation to fasting is dependent on peroxisome proliferator-activated receptor alpha transcriptional up-regulation of fatty acid oxidation. Endocrinology (2005) 146(1):375–82. 10.1210/en.2004-0667 15459119

[109] Fajans SS Bell GI Macrosomia and neonatal hypoglycaemia in RW pedigree subjects with a mutation (Q268X) in the gene encoding hepatocyte nuclear factor 4α (HNF4A). Diabetologia (2007) 50(12):2600–1. 10.1007/s00125-007-0833-7 17891372

[110] Kapoor RR Locke J Colclough K Wales J Conn JJ Hattersley AT Persistent hyperinsulinemic hypoglycemia and maturity-onset diabetes of the young due to heterozygous HNF4A mutations. Diabetes (2008) 57(6):1659–63. 10.2337/db07-1657 18268044

[111] Flanagan SE Kapoor RR Mali G Cody D Murphy N Schwahn B Diazoxide-responsive hyperinsulinemic hypoglycemia caused by HNF4A gene mutations. Eur J Endocrinol (2010) 162(5):987–92. 10.1530/eje-09-0861 20164212 PMC2857991

[112] Thomas H Jaschkowitz K Bulman M Frayling TM Mitchell SMS Roosen S A distant upstream promoter of the HNF-4α gene connects the transcription factors involved in maturity-onset diabetes of the young. Hum Mol Genet (2001) 10(19):2089–97. 10.1093/hmg/10.19.2089 11590126

[113] Rozenkova K Malikova J Nessa A Dusatkova L Bjorkhaug L Obermannova B High incidence of heterozygous ABCC8 and HNF1A mutations in czech patients with congenital hyperinsulinism. J Clin Endocrinol Metab (2015) 100(12):E1540–9. 10.1210/jc.2015-2763 26431509

[114] Otonkoski T Jiao H Kaminen-Ahola N Tapia-Paez I Ullah MS Parton LE Physical exercise–induced hypoglycemia caused by failed silencing of monocarboxylate transporter 1 in pancreatic β cells. Am J Hum Genet (2007) 81(3):467–74. 10.1086/520960 17701893 PMC1950828

[115] Meissner T Otonkoski T Feneberg R Beinbrech B Apostolidou S Sipila I Exercise induced hypoglycaemic hyperinsulinism. Arch Dis Child (2001) 84(3):254–7. 10.1136/adc.84.3.254 11207177 PMC1718690

[116] Tu N Chen H Winnikes U Reinert I Marmann G Pirke KM Structural organization and mutational analysis of the human uncoupling protein-2 (hUCP2) gene. Life Sci (1999) 64(3):l41–50. 10027754 10.1016/s0024-3205(98)00555-4

[117] Krauss S Zhang C-Y Lowell BB A significant portion of mitochondrial proton leak in intact thymocytes depends on expression of UCP2. Proc Natl Acad Sci U S A (2002) 99(1):118–22. 10.1073/pnas.012410699 11756659 PMC117524

[118] González-Barroso MM Giurgea I Bouillaud F Anedda A Bellanné-Chantelot C Hubert L Mutations in *UCP2* in congenital hyperinsulinism reveal a role for regulation of insulin secretion. PLoS One (2008) 3(12):e3850. 10.1371/journal.pone.0003850 19065272 PMC2588657

[119] Hojlund K Hansen T Lajer M Henriksen JE Levin K Lindholm J A novel syndrome of autosomal-dominant hyperinsulinemic hypoglycemia linked to a mutation in the human insulin receptor gene. Diabetes (2004) 53(6):1592–8. 10.2337/diabetes.53.6.1592 15161766

[120] Samuk I Afriat R Horne T Bistritzer T Barr J Vinograd I. Dumping syndrome following Nissen fundoplication, diagnosis, and treatment. J Pediatr Gastroenterol Nutr (1996) 23(3):235–40. 10.1097/00005176-199610000-00006 8890072

[121] Palladino AA Sayed S Levitt Katz LE Gallagher PR De Leon DD. Increased glucagon-like peptide-1 secretion and postprandial hypoglycemia in children after Nissen fundoplication. J Clin Endocrinol Metab (2009) 94(1):39–44. 10.1210/jc.2008-1263 18957502 PMC2630870

[122] Kneepkens CM Fernandes J Vonk RJ. Dumping syndrome in children. Diagnosis and effect of glucomannan on glucose tolerance and absorption. Acta Paediatr Scand (1988) 77(2):279–86. 10.1111/j.1651-2227.1988.tb10643.x 2833060

[123] Basu A Service FJ Yu L Heser D Ferries LM Eisenbarth G. Insulin autoimmunity and hypoglycemia in seven white patients. Endocr Pract (2005) 11(2):97–103. 10.4158/EP.11.2.97 15901524

[124] Lupsa BC Chong AY Cochran EK Soos MA Semple RK Gorden P. Autoimmune forms of hypoglycemia. Medicine (2009) 88(3):141–53. 10.1097/MD.0b013e3181a5b42e 19440117

[125] Chandarana K Batterham RL. Shedding pounds after going under the knife: metabolic insights from cutting the gut. Nat Med (2012) 18(5):668–9. 10.1038/nm.2748 22561824

[126] Yousseif A Emmanuel J Karra E Millet Q Elkalaawy M Jenkinson AD Differential effects of laparoscopic sleeve gastrectomy and laparoscopic gastric bypass on appetite, circulating acyl-ghrelin, peptide YY3-36 and active GLP-1 levels in non-diabetic humans. Obes Surg (2014) 24(2):241–52. 10.1007/s11695-013-1066-0 23996294 PMC3890046

[127] Service GJ Thompson GB Service FJ Andrews JC Collazo-Clavell ML Lloyd RV. Hyperinsulinemic hypoglycemia with nesidioblastosis after gastric-bypass surgery. N Engl J Med (2005) 353(3):249–54. 10.1056/NEJMoa043690 16034010

[128] Service FJ Natt N Thompson GB Grant CS van Heerden JA Andrews JC Noninsulinoma pancreatogenous hypoglycemia: a novel syndrome of hyperinsulinemic hypoglycemia in adults independent of mutations in Kir6.2 and SUR1 genes. J Clin Endocrinol Metab (1999) 84(5):1582–9. 10.1210/jc.84.5.1582 10323384

[129] Won JG Tseng HS Yang AH Tang KT Jap TS Lee CH Clinical features and morphological characterization of 10 patients with noninsulinoma pancreatogenous hypoglycaemia syndrome (NIPHS). Clin Endocrinol (2006) 65(5):566–78. 10.1111/j.1365-2265.2006.02629.x 17054456

[130] Ashcroft FM. ATP-sensitive potassium channelopathies: focus on insulin secretion. J Clin Invest (2005) 115(8):2047–58. 10.1172/jci25495 16075046 PMC1180549

[131] Service FJ McMahon MM O’Brien PC Ballard DJ Functioning insulinoma – incidence, recurrence, and long-term survival of patients: a 60-year study. Mayo Clin Proc (1991) 66(7):711–9. 10.1016/S0025-6196(12)62083-7 1677058

[132] Kar P Price P Sawers S Bhattacharya S Reznek RH Grossman AB. Insulinomas may present with normoglycemia after prolonged fasting but glucose-stimulated hypoglycemia. J Clin Endocrinol Metab (2006) 91(12):4733–6. 10.1210/jc.2006-1430 17003090

[133] Cryer PE Axelrod L Grossman AB Heller SR Montori VM Seaquist ER Evaluation and management of adult hypoglycemic disorders: an Endocrine Society Clinical Practice Guideline. J Clin Endocrinol Metab (2009) 94(3):709–28. 10.1210/jc.2008-1410 19088155

